# NMR spectroscopy derived plasma biomarkers of inflammation in human populations: Influences of age, sex and adiposity

**DOI:** 10.1371/journal.pone.0311975

**Published:** 2025-01-06

**Authors:** Samantha Lodge, Reika Masuda, Philipp Nitschke, John P. Beilby, Jennie Hui, Michael Hunter, Bu B. Yeap, Oscar Millet, Julien Wist, Jeremy K. Nicholson, Elaine Holmes

**Affiliations:** 1 Australian National Phenome Center and Center for Computational and Systems Medicine, Health Futures Institute, Murdoch University, Perth, Western Australia, Australia; 2 School of Biomedical Sciences, University of Western Australia, Perth, Western Australia, Australia; 3 PathWest Laboratory Medicine, Queen Elizabeth II Medical Centre, Perth, Western Australia, Australia; 4 School of Population and Global Health, University of Western Australia, Perth, Western Australia, Australia; 5 School of Medicine, University of Western Australia, Crawley, Western Australia, Australia; 6 Department of Endocrinology and Diabetes, Fiona Stanley Hospital, Murdoch, Western Australia, Australia; 7 Precision Medicine and Metabolism Laboratory, CIC bioGUNE, Parque Tecnológico de Bizkaia, Derio, Spain; 8 Department of Metabolism, Digestion and Reproduction, Faculty of Medicine, Imperial College London, London, United Kingdom; 9 Chemistry Department, Universidad del Valle, Cali, Colombia; 10 Institute of Global Health Innovation, Faculty of Medicine, Imperial College London, London, United Kingdom; Catholic University of Brasilia, BRAZIL

## Abstract

Understanding the distribution and variation in inflammatory markers is crucial for advancing our knowledge of inflammatory processes and evaluating their clinical utility in diagnosing and monitoring acute and chronic disease. ^1^H NMR spectroscopy of blood plasma and serum was applied to measure a composite panel of inflammatory markers based on acute phase glycoprotein signals (GlycA and GlycB) and sub-regions of the lipoprotein derived Supramolecular Phospholipid Composite signals (SPC_1_, SPC_2_ and SPC_3_) to establish normal ranges in two healthy, predominantly white cohorts from Australia (n = 398) and Spain (n = 80; ages 20–70 years). GlycA, GlycB, SPC_1_ and SPC_3_ were not significantly impacted by age or sex, but SPC_2_ (an HDL-related biomarker) was significantly higher in women across all age ranges by an average of 33.7%. A free-living Australian population cohort (n = 3945) was used to explore the relationship of BMI with the panel of inflammatory markers. The glycoprotein signals were directly associated with BMI with GlycB levels being significantly higher for women in all BMI classes. Conversely, SPC_2_ was found to be inversely associated with BMI and differed significantly between the sexes at each BMI category (normal weight *p* = 3.46x10^-43^, overweight *p* = 3.33x10^-79^, obese *p* = 2.15x10^-64^). SPC_1_ and SPC_3_ were markedly less affected by BMI changes. Given the significant association between SPC_2_ and sex, these data suggest that men and women should be modelled independently for NMR-determined inflammatory biomarkers, or that data should be corrected for sex.

## Introduction

Inflammation is an important component of the body’s normal generic defence mechanism to injury or pathogens and triggers a cascade of events to maintain organ and whole-body homeostasis [1]. However, wherein acute inflammation is a necessary immediate adaptive response to injury (tissue damage) or infection, chronic inflammation which persists after the initial event, or results from chronic stress or autoimmune disorders, has the potential to induce local or systemic long term tissue damage and disease [2].

Chronic inflammatory diseases have been recognised as the leading cause of mortality worldwide. Diseases that have substantial inflammatory components include stroke, cancer, cardiovascular disease, diabetes mellitus and autoimmune diseases such as rheumatoid arthritis [3, 4]. Systemic chronic inflammation can impair normal immune function, leading to a greater frequency of infections [5, 6]. In addition, it has been shown that chronic inflammation during pregnancy and childhood leads to elevated risk of non-communicable diseases later in life [7, 8]. Thus, there is a critical need to understand and manage inflammation. The clinical gold standard metric for identifying and monitoring inflammation is blood serum C-reactive protein (CRP). CRP is an acute phase protein that is synthesised by hepatocytes in response to secretion of pro-inflammatory cytokines by macrophages and adipocytes [9]. Other inflammatory markers such as α-1-acid glycoproteins have also been found to be robust markers of inflammation and in one study comparing the performance of CRP and an α-1-acid glycoprotein signal, measured using nuclear magnetic resonance (NMR) spectroscopy (GlycA), CRP was more closely associated with obesity, whereas GlycA was a better risk predictor for cardiovascular disease [10].

Acute phase N-acetylated glycoproteins can be efficiently quantified using ^1^H NMR spectroscopy in the form of observed signals, GlycA and GlycB [11]. These are composite overlapped NMR signals where GlycA has contributions from α-1-acid glycoprotein, α1-antitrypsin, α1-antichymotrypsin and haptoglobin, whereas the GlycB signal has contributions from the *N*-acetyl methyl groups of N-acetylneuraminic acid residues in the glycoproteins [12]. The GlycA and GlycB NMR signals become elevated in chronic and acute inflammation and one study has suggested that these markers are more reliable than high sensitivity C-reactive protein (hsCRP) in monitoring acute infectious disease episodes [13, 14]. Using diffusion and T_2_ relaxation edited (DIRE) ^1^H spectroscopy [11, 15] to quantify these glycoproteins, an additional peak was discovered in the ^1^H NMR spectrum at 3.20–3.30 ppm. The signal originates from choline headgroups (^+^N-(CH_3_)_3_) of phospholipids [16] compartmentalised in lipoproteins that have specific diffusional and relaxation properties that allow them to be characterised and quantified by specific NMR pulse sequences [11]. This broad composite peak was named the Supramolecular Phospholipid Composite (SPC) and was found to be markedly reduced in acute SARS-CoV-2 patients in comparison to healthy control participants [11] as well as in participants with chronic inflammation, for example in children 3 years post burn injury [17]. Further characterization of the composite peak revealed it was comprised of three distinct regions, SPC_1_, SPC_2_ and SPC_3_, each associated with different lipoprotein subfractions. SPC_1_ is the high-density lipoprotein (HDL) containing phospholipid subfraction 4 (the smallest, highest density of the HDL subfractions), SPC_2_ is the signal associated with lipoprotein subfractions HDL1, HDL2 and HDL3, and SPC_3_ is associated with low density lipoprotein (LDL) [18].

Further analysis of the SPC signal indicated a differential behaviour between the sub-regions under different pathological conditions. Ryan *et al* showed significant decreases of SPC_1_ in acute burn injury, which persisted at the 6 week follow up, while SPC_3_ showed no significant difference at the time of burn injury but was significantly different from healthy controls at 6 week follow up [19]. A further study on non-septic patients demonstrated a differential time scale change between the glycoproteins and SPC following admission into ICU, wherein at admission, the SPC subregions but not the glycoproteins, were significantly different from healthy controls. However, at 48 hours post admission, the glycoproteins and SPC were both significantly different between patients and controls indicating that SPC demonstrates a more rapid response to acute injury than the Glyc signal [20]. In addition, SPC, which increases throughout pregnancy has also been shown to be inversely associated with alignment to the mediterranean diet in a maternal pregnancy cohort [21].

The rising number of individuals with chronic inflammatory diseases and the associated economic burden demand deeper understanding of the aetiopathology of inflammation with refined biomarker panels that can more accurately indicate the inflammatory status of an individual. Thus, there is an imperative for full characterisation of the new inflammatory marker, SPC and for establishing normal population blood concentration ranges. The use of a modified NMR pulse sequence allows inflammatory signals GlycA, GlycB and SPC to be accurately quantified. There are increasing numbers of publications using SPC as an inflammatory marker [22, 23], where studies have shown that a reduction in the SPC signal occurs when inflammation is present, often differentially between the signal sub-regions. However, to date there are no studies that have determined the expected ranges of the SPC sub-regions in individuals considered normal or healthy. To explore this, we combined data deriving from samples from two independent cohorts, (a) a population-based study consisting of 1976 participants at two time points, sampled 5 years apart [24], (b) control samples (n = 80) collected by the Basque Biobank for research (BIOEF). Here we sought to determine the expected ranges of GlycA, GlycB, SPC_1_, SPC_2_ and SPC_3_ in healthy individuals. In addition, we explore the impact of sex and BMI on these inflammatory markers.

## Materials and methods

### Participant enrolment and sample collection

Participants were included from two distinct cohort studies.

#### Population cohort (Busselton Healthy Ageing Study)

The Busselton Healthy Ageing Study (BHAS) is a community-based, prospective cohort study involving adults born between 1946–1964, resident in the Busselton Shire, Western Australia [25]. A total of 1976 participants were included in this study with samples obtained at two time points (n = 3945). These participants underwent the first blood collection during the first wave of the study in 2010–2015 and had repeat collections in the second wave from 2016–2021. The University of Western Australia’s Human Research Ethics Committee (2021/ET000260) and Murdoch University Ethics Committee (2020/132) approved the study and all participants provided written consent. Participants completed questionnaires detailing sociodemographic and lifestyle information. Height, weight, and blood pressure were measured and routine haematological and biochemical testing on blood samples were performed as previously described [25]. The complete demographic data collected can be found in S1 Table in S1 File.

#### Basque population (Biogune) cohort

The cohort consisted of 80 healthy control participants. All samples were collected by the Basque Biobank for research (BIOEF) at the Cruces University Hospital (Barakaldo, Spain) from 2019–2020. All participants provided informed written consent to clinical investigations, according to the Declaration of Helsinki, and all data were anonymized to protect their confidentiality. The sample handling protocol was evaluated and approved by the *Comité de Ética de Investigación con medicamentos de Euskadi* (CEIm-E, PI+CES-BIOEF 2020–04 and PI219130). Shipment of human samples to the Australian National Phenome Centre (ANPC) had the approval of the Ministry of Health of the Spanish Government. All samples were approved for analysis as part of the International Severe Acute Respiratory and Emerging Infection Consortium (ISARIC) / World Health Organization (WHO) pandemic trial framework (SMHS Research governance office PRN:3976 and Murdoch University Ethics no 2020/052). The full cohort demographic collected can be found in S2 Table in S1 File. These participants have been previously used as healthy controls within other studies [20, 26].

### Definition of ‘healthy’ participants

All individuals from the Basque cohort were considered healthy. From the population-based cohort individuals were only selected where individuals were non-smokers, did not have any medical conditions as listed in the respective population cohort methods, had normal blood pressure and a BMI<30 kg/m^2^. These selection criteria have been described previously [24].

### ^1^H NMR sample preparation

Serum and plasma samples were stored at -80°C until the day of analysis, when they were defrosted at room temperature for 30 minutes. Each sample was prepared as a mixture of phosphate buffer (75 mM Na_2_HPO_4_, 2 mM NaN_3_, 4.6 mM sodium trimethylsilyl propionate-[2,2,3,3-^2^H_4_] (TSP) in H_2_O/D_2_O 4:1, pH 7.4 ±0.1) and serum or plasma at a 1:1 ratio for a final volume of 600 μL into the 5 mm SampleJet^TM^ NMR tubes. Samples were then manually shaken for several seconds and stored at 5°C inside the SampleJet^TM^ automatic sample changer until measurement (< 24h).

### ^1^H NMR spectroscopy data acquisition and processing parameters

**Fig 1:**
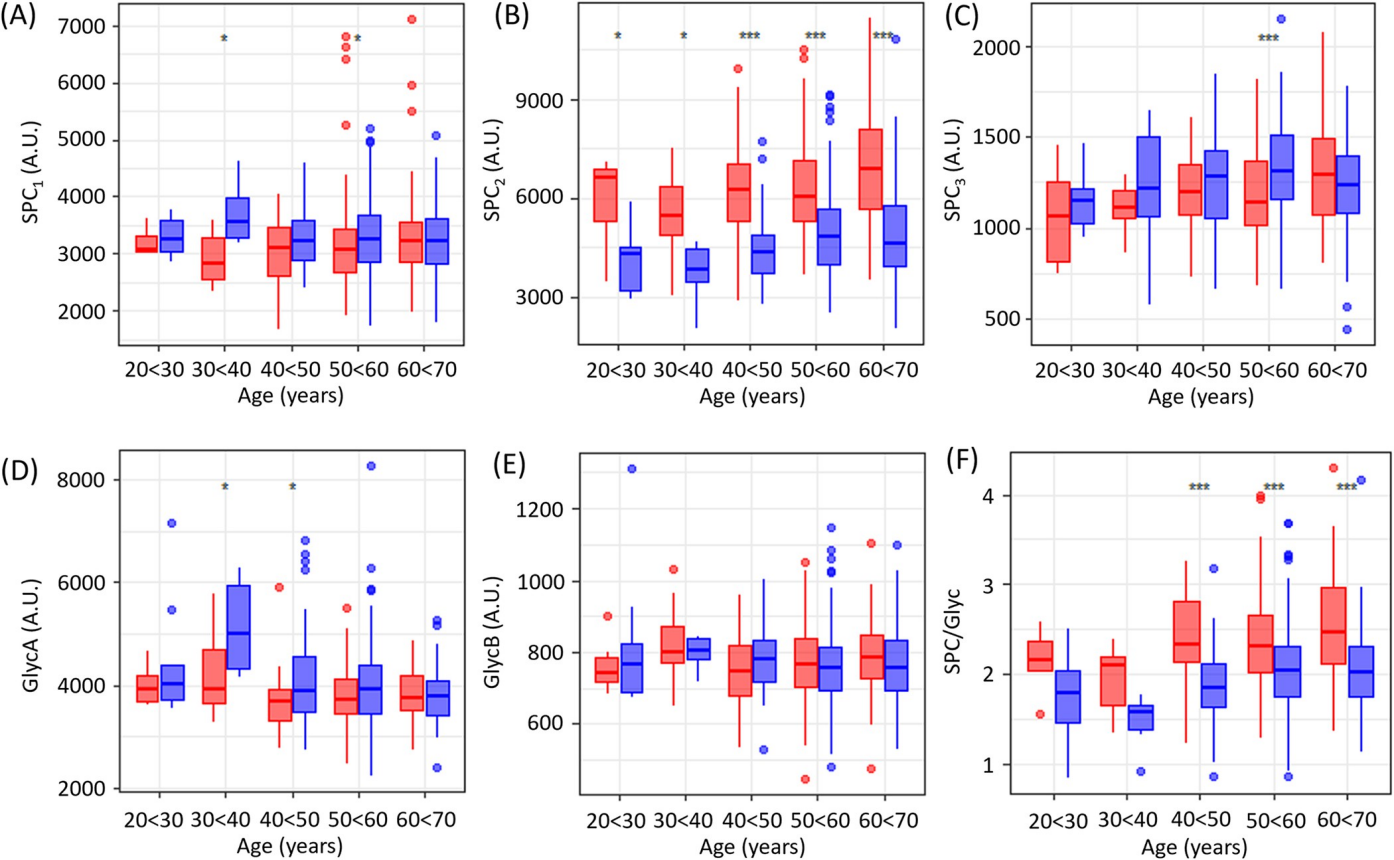
Box plots showing age and sex (blue: male, red: female) differences in healthy individuals of (A) SPC_1_ (B) SPC_2_ (C) SPC_3_ (D) GlycA (E) GlycB and (F) SPC_total_/Glyc. Only the significant differences between males and females at each age range are highlighted using the *p*-value (*p<0.05; **p<0.01; ***p<0.001). All *p*-values can be found in S3 Table in S1 File.

NMR spectroscopic analyses were performed on 600 MHz Bruker Avance III HD spectrometers, equipped with a 5 mm BBI probe and fitted with the Bruker SampleJet^TM^ robot cooling system set to 5°C. A full quantitative calibration was completed prior to sample measurement using a protocol described elsewhere, where the ERETIC peak is at 15 ppm [27]. For each sample two experiments were performed to measure the proton nuclei, both completed at 310K. A single DIRE experiment (64 scans, 98k data points, a spectral width of 30 ppm with a total experiment time of 4 min 25 s) with a continuous secondary irradiation field at the water frequency for solvent suppression to obtain the inflammatory markers [11]. And a single one-dimensional (1D) NMR experiment using the Bruker In Vitro Diagnostics research (IVDr) methods where the standard 1D experiment was performed with solvent presaturation (32 scans, 98k data points, spectral width of 30 ppm, and experiment time of 4 minutes). This was performed to quantify the high- and low-density lipoproteins containing phospholipids using the Bruker IVDr Lipoprotein Subclass Analysis (B.I.LISA) method. The B.I.LISA method integrates the -CH_3_ and -CH_2_- groups from the lipoproteins at the chemical shifts of 0.80 and 1.25 ppm and fits them using a PLS-2 regression model. The density of LDL is 1.019–1.063 kg/L and HDL is 1.063–1.210 kg/L. The HDL subfractions are classified into four density classes as HDL-1 (1.063–1.100 kg/L), HDL2 (1.100–1.125 kg/L), HDL3 (1.125–1.175 kg/L), and HDL4 (1.175–1.210 kg/L) [28]. Lodge et al, Fig 1 shows the full integrated NMR spectrum, clearly labelled with compound name and chemical structure [11]. NMR analyses of the population cohort was completed January-June 2021, and the Basque population cohort was completed in December 2020.

### Data processing

All NMR data processing was completed in R using in-house developed open-source packages “nmr-parser” and “nmr-spectra-processing” [29, 30]. All DIRE spectral references were calibrated to 0 (SR = 0), that is equivalent to calibration to the water suppression frequency. All data points of the DIRE spectra were corrected for quantification to account for variability within the instrument using the ERETIC factor (this is saved in the XML file called “QuantFactorSample” for each sample). The DIRE spectrum was baseline corrected using an asymmetric least-squares routine; spectral regions containing the residual water peak (δ 4.50−4.90) and regions containing predominantly noise (δ < 0.4 and δ > 9.5) were excluded from the analyses. The integration of GlycA, GlycB and the SPC region was achieved by summation of a fixed spectral region (GlycA: δ 2.05−2.09, GlycB: δ 2.09−2.12, SPC_1_ δ 3.20−3.236, SPC_2_ δ 3.236−3.262; and SPC_3_ δ 3.262−3.30) after preprocessing.

### Data analysis

All computation and data visualisation were performed using R and RStudio with the open-source R package “*metabom8*” (version 0.2), available from GitHub (github.com/tkimhofer/metabom8). Data were adjusted for sex, where stated in the results section, using R package “*nlme”*. After correction the mean of each unadjusted variable was added onto the adjusted values. For univariate comparisons, Mann-Whitney rank sum tests were performed. Adjusted *p*-values were determined using the method proposed by Benjamini and Hochberg [31]. Analysis of the data for this study was conducted from June 2023-April 2024.

## Results and discussion

### Characterisation of healthy individuals—age and sex differences

The healthy participants were selected from the Basque cohort and the BHAS cohort and included 478 participants (271 male; 207 female) non-smokers with no reported medical conditions, non-smokers and who were not obese (BMI<30 kg/m^2^). In addition, for the BHAS cohort only those that self-rated their health as “good”, “very good” or “excellent” were included. Previously, selection of healthy participants in the BHAS cohort has been published, together with comparisons of lipoprotein profiles in healthy participants versus those with different chronic conditions [24].

It is notable that the current terminology used for the NMR-generated lipoprotein data defines HDL1 as 1.063–1.100 g/ml, HDL2 as 1.100–1.112 g/ml, HDL3 as 1.112–1.125 g/ml and HDL4 as 1.125–1.21 g/ml, whereas previous literature based on ultracentrifugation and electrophoresis use the terminology H2 to reflect the density range (1.063–1.125 g/ml or 2a 1.100–1.125 g/ml and 2b 1.063–1.100 g/ml) and H3 to reflect the density range (1.125–1.21 g/ml) [32]. For the remainder of this article any reference to literature will refer to the results according to the HDL1-4 categories established for NMR spectroscopy [28].

The sex differences of healthy individuals were compared for different age groups (20–29; 30–39; 40–49; 50–59, 60–70 years; Fig 1). Distributions of SPC_1_ (Fig 1A) and SPC_3_ (Fig 1C), while having a predominantly larger median value in the males, were largely comparable between the sexes for all age groups. In the case of SPC_2_, values were significantly higher in women than men across all age categories: for example, the *p*-value, between male and females in the age range 50–60 years was 7.87x10^-12^ (S3 Table in S1 File). SPC_2_ (Fig 1B) is known to represent the phospholipid components in HDL1-3 [18]. When comparing HDL distributions between the sexes, several studies have reported higher basal levels of total HDL1-3 and HDL1-3 cholesterol (density 1.063–1.125 g/ml) in women [23, 33, 34] in agreement with the findings here. Although differences in HDL1-3 concentrations have been reported for different ethnicities, with lower reference ranges in African populations in comparison to white populations, both the total HDL cholesterol and higher concentrations of HDL1-3 cholesterol are consistent across different populations [33]. The higher HDL2 cholesterol concentrations in women are thought to be mediated partially by the age-associated, estrogen-induced downregulation of hepatic lipase, which serves to degrade HDL1-3 and partially by the capacity of estrogens to increase apolipoprotein A1 [35]. Rossler *et al* reported no correlation with age based on the total SPC peak total intensity but did not stratify for sex [36]. Similarly, for SPC_2_ where we observed higher levels in women, SPC total was always significantly higher in women than in men in each age range (S1 Fig in S1 File). As this sex-dependent pattern was not observed for SPC_1_ or SPC_3_, and since SPC_2_ is ca 5-fold greater than SPC_3_ and 2-fold greater than SPC_1_, it is likely that SPC_2_ is responsible for driving the sex differences in the calculated concentration for SPC total.

When comparing age differences for SPC levels in healthy men and women separately, no significant differences were observed (S2 and S3 Figs in S1 File). This is coherent with reports that the phospholipid content of total HDL does not differ between young and elderly individuals, which is consistent with the lack of impact of aging on the cholesterol efflux capacity of HDL [37]. In contrast, Richter et al reported that serum HDL1-3 cholesterol levels declined with age in women, whereas HDL1-3 triglycerides increased in accordance with a decrease in post heparin lipolysis capacity [38]. In a separate study by Frey et al, phospholipids in HDL1-3 subfractions were found to be lower in older and less active individuals [39]. Similarly, aging was shown to decrease the phospholipid content of both HDL1-3 (associated with SPC_2_) and HDL4 (associated with SPC_1_) and also resulted in a reduction in the fluidity of the phospholipidic layer membrane in HDL in elderly individuals [40].

Median values of GlycA were higher for men than women in the 30<40 and 40<50 years age strata (Fig 1D), however, statistical power is low when the dataset is stratified for age and sex, requiring further larger cohorts for validation. Serum concentrations of GlycB were not differentiated by sex (Fig 1E). When comparing healthy men and women separately, healthy men aged 30<40 had higher GlycA than those aged 60<70, with no significant differences in healthy women, and no differences observed for GlycB levels across any age groups (S2 and S3 Figs in S1 File). Whilst several studies have reported that serum GlycA levels are higher in women than men [41, 42], Ballout et al argue that the difference in the GlycA levels between sexes is less than 10% and therefore, unlike serum or plasma CRP concentrations, which are substantially higher in women, GlycA does not demonstrate strong sex dependency [43]. It has previously been shown that GlycA is positively associated with age in familial hypercholesterolemia patients [42]. However, studies have yet to determine an association with age in a healthy population. It was found that the SPC/Glyc ratio (Fig 1F) was higher in women than men from age 40–70 years, but no significant differences were observed at the younger ages, likely due to low statistical power in those strata. The median and ranges of the SPC and Glyc for men and women can be found in S4 and S5 Tables in S1 File.

### Age, sex, and BMI strongly impact the level of SPC_2_ but SPC_1_ and SPC_3_ are much less affected

**Fig 2:**
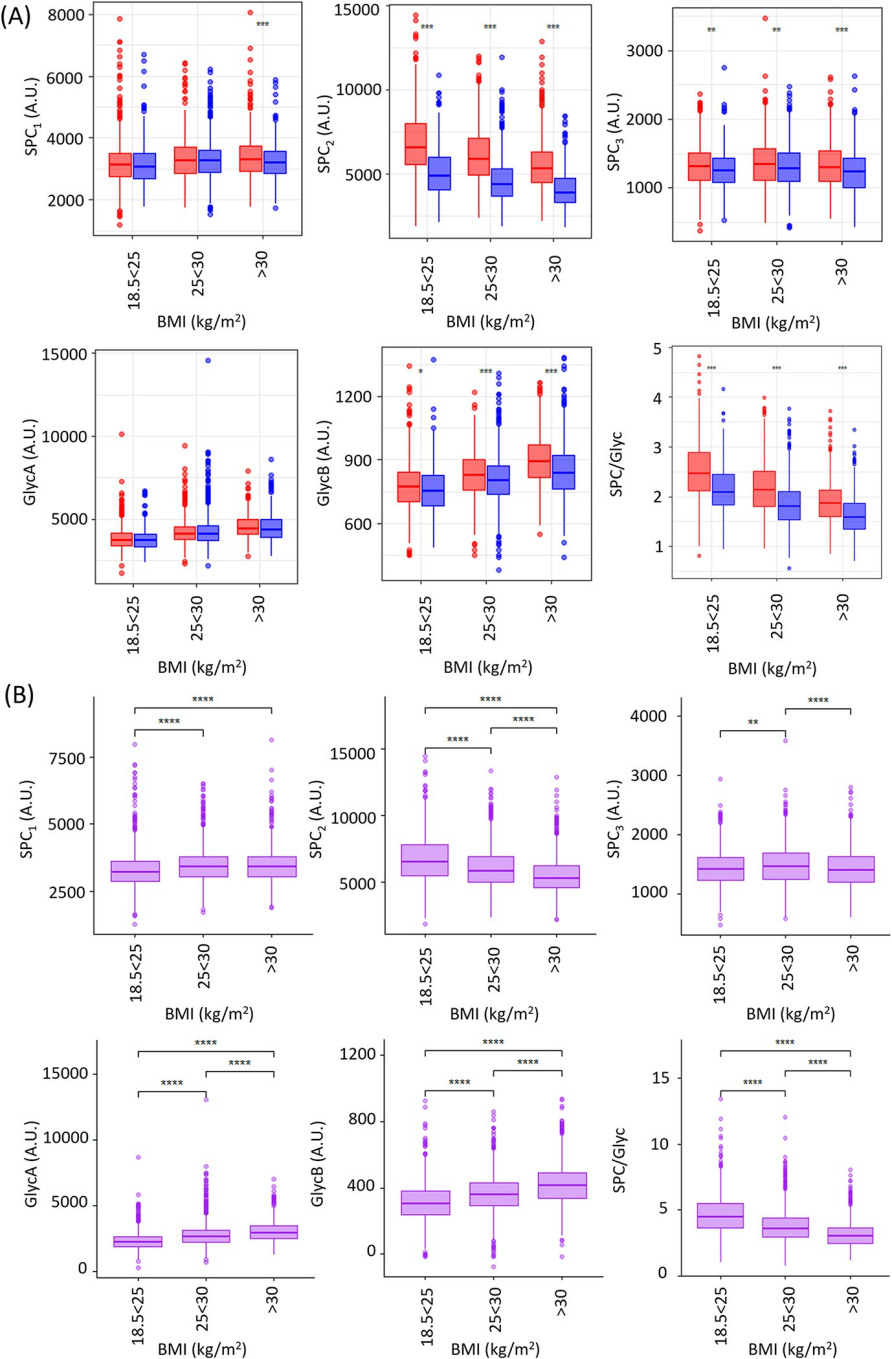
(A) Box plots showing BMI and sex (blue: male, red: female) differences for SPC_1_, SPC_2_, SPC_3_, GlycA, GlycB and SPC/Glyc in the population cohort (n = 3,936, 9 underweight individuals removed from this analysis). Only the significant differences between men and women at each age range are highlighted using the *p*-value (*p<0.05; **p<0.01; ***p<0.001). (B) Box plots showing BMI differences for SPC_1_, SPC_2_, SPC_3_, GlycA, GlycB and SPC/Glyc in the population cohort where correction for sex has been applied. Only the significant differences between men and women at each age range are highlighted using the *p*-value (*p<0.05; **p<0.01; ***p<0.001, ****p<0.0001). All adjusted p-values can be found in S6 and S8 Tables in S1 File.

Having observed systematically higher serum concentrations of SPC_2_ in the sub-cohort of healthy women, the free-living population cohort was used to determine the association of BMI with the SPC and Glyc signals in a broader population. In the first instance sex variation was investigated for SPC (Fig 2A). There were highly significant differences in the distributions of SPC_2_ and, to a lesser extent SPC_3_, between men and women at every BMI range: normal weight (BMI 18.5<25 kg/m^2^), overweight (BMI 25<30 kg/m^2^) and obese (BMI >30 kg/m^2^) (Fig 2A and S6 Table in S1 File), where the median values were higher for women across all BMI categories (S7 Table in S1 File). Interestingly, SPC_1_ showed no sex differences in the normal and overweight categories but significant differences for the obese stratum (*p* = 3.67x10^-4^) (Fig 2A). However, the difference in median values between men and women for SPC_2_ (normal weight: men 5123.65 A.U., women 6837.86 A.U.; overweight men 4609.95 A.U., women 6136.34 A.U.; obese men 4085.73 A.U., women 5502.43 A.U.) were far greater than for SPC1 (obese men 3221.94 A.U., women 3362.88 A.U.) or for SPC_3_. When considering male and female cohorts separately, SPC_2_ was significantly different for both sexes across all BMI ranges (S4 and S5 Figs in S1 File). For SPC_3_ there was no significant difference across the BMI ranges for women, while for the male subset there were significant differences between the healthy BMI versus overweight BMI (*p* = 3.90x10^-2^) and for the overweight versus obese BMI groups (*p* = 1.60x10^-5^).

Unlike the healthy control subset where the GlycA levels were higher for men than women (Fig 1D), in the population as a whole, GlycA demonstrated no significant differences between men and women, whereas GlycB was significantly different between the sexes being higher in women than men in each BMI class (Fig 2A). Moncayo et al reported higher GlycB values in women with polycystic ovary syndrome compared to men [44]. When comparing the males and female cohorts separately, there were significant differences between all the BMI classes for both GlycA and GlycB (S4 and S5 Figs in S1 File). To further interrogate BMI effects on the inflammatory markers the data were corrected for sex (Fig 2B). In this sex-adjusted dataset, SPC_2_ was inversely associated with BMI, whereas GlycA and GlycB were significantly higher with each incremental increase in class of BMI. SPC_1_ was not significantly different between the overweight BMI and obese BMI classes.

As an individual becomes overweight and/or subsequently obese, adipose tissue releases inflammatory mediators, for example interleukin 6 and tumour necrosis factor α (TNF-α) and reduces production of adiponectin [45]. Chronic inflammation has been associated with obesity [46], but often clinical symptoms of inflammation are absent. It has been shown that BMI has a threshold effect for chronic inflammation as measured using hsCRP, wherein a BMI up to 24.3 is not correlated with chronic inflammation, whereas a BMI greater than 24.3 is positively correlated with chronic inflammation [47]. It was also observed that the pro-inflammatory effect caused by BMI was stronger in women, consistent with findings herein. GlycA and B have been shown to correlate strongly with the particle numbers of triglyceride rich lipoproteins in fatty liver disease, unlike hsCRP, which did not show such a strong correlation [48], implying that the Glyc signature reports on a different aspect of the inflammatory processes. Other literature cites significant direct association between GlycA and obesity [49, 50] with consequent reduction in GlycA following dietary or surgical weight loss interventions [51, 52]. The significant differences between these inflammatory markers, with SPC_2_ showing a strong inverse and GlycA and GlycB a direct association with BMI class, drives highly significant differences in the SPC/Glyc ratio between classes of BMI, such that the ratio is higher in women than men, and is inversely associated with BMI in sex-adjusted analyses (Fig 2A and 2B).

**Fig 3:**
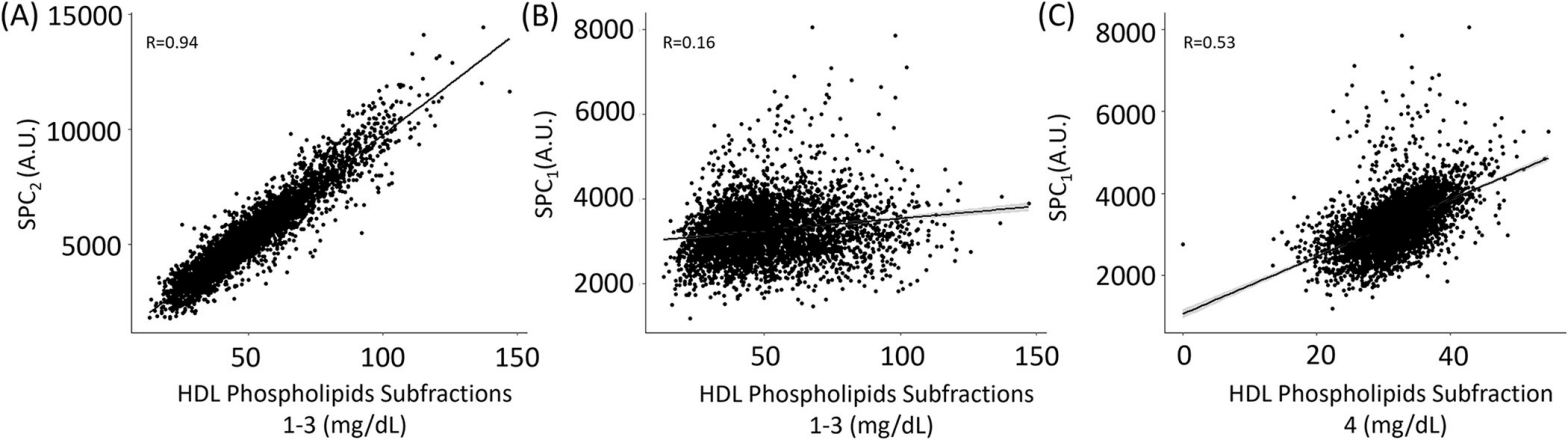
Linear correlation models of (A) SPC_2_ versus HDL phospholipids subfractions 1–3 (r = 0.94) (B) SPC_1_ versus HDL phospholipids subfractions 1–3 (r = 0.16) and (C) SPC_1_ versus HDL phospholipids subfraction 4 (r = 0.53). (n = 4025, BHAS and Basque population included).

To date, this is the first study to report BMI-related differences for each of the three distinct SPC regions. However, a previous study demonstrated that SPC total decreases as BMI increases [36]. This is unsurprising when considering the signal intensity of each SPC region; SPC_2_ dominates the signal profile and is often more than twice as large as SPC_1_ and five times as large as SPC_3_. When SPC_2_ is inversely associated with BMI, this drives the overall correlation between the composite SPC peak and BMI effect. Indeed, when comparing SPC distributions there is a significant difference between men and women for each BMI class (S6 Fig in S1 File), where the *p*-value for those considered a healthy weight was (1.19x10^-35^), overweight (1.26x10^-60^) and those that are obese (2.49x10^-53^). The inverse association of SPC with BMI was consistent for both sexes, despite women having higher concentrations of SPC in each class of BMI (S7A and S7B Fig in S1 File). However, we have shown here that the 3 different SPC regions behave differently as each is associated with different lipoprotein subfractions. In keeping with the observed differences in the SPC regions with respect to sex, age and BMI, correlation of the individual SPC regions with the different HDL fractions show very different correlation coefficients (Fig 3 and S8 Fig in S1 File). These results are consistent with SPC_1_ reflecting the smaller and denser HDL lipoprotein subfraction 4, which returns a correlation coefficient of r = 0.53 between SPC_1_ and HDL4 for the full BHAS and Basque cohorts, whereas the correlation with HDL1-3 subfractions reflecting was much lower (r = 0.16). The most striking correlation is between HDL1-3 (density 1.063–1.125 g/ml) and SPC_2_ (r = 0.94), which is consistent with the inverse correlations found between HDL1-3 and obesity in the literature [53], whereas most of these studies did not find the same relationship for HDL4 and obesity [54, 55]. Although subparticles HDL1-4 have been shown to be low in coronary artery disease compared to controls, there was a proportionally larger reduction in HDL1-3 cholesterol and phospholipids subfractions compared to those in the HDL4 subfraction [56].

### Considerations for application of the NMR-derived inflammatory panel

Although the combined population of the BHAS and the Basque cohort are well powered (n = 4025 samples), the number of ‘healthy’ participants, as defined by non-smokers with normal blood pressure, a BMI<30 kg/m^2^ and as individuals who did not have any medical conditions was much lower (n = 478), as would be expected in a predominantly white population with a western lifestyle. A recent survey from the Australian Bureau of statistics reported that 81.4% of Australian adults had at least one chronic health condition [57]. This may account for some of the disparities between the healthy and the full population. For example, healthy women and men have comparable serum GlycB concentrations, but in the full population women in all BMI categories have higher concentrations of GlycB, which is consistent with literature reports and with women having generally higher levels of hsCRP. Thus, it is important to take the background of the cohort into account.

We, and others have shown strong correlation between obesity and GlycA and GlycB values supporting the underlying role of acute phase glycoproteins in inflammation [49, 52]. NMR now offers a rapid and robust means of measuring these parameters, using the DIRE NMR pulse sequence [11], thereby removing the contributions from proteins such as albumin [58]. In terms of the SPC composite signal, we have clearly shown that SPC_1_, SPC_2_ and SPC_3_ behave very differently in terms of their association with age, sex and BMI. This cautions the use of the SPC signal as a single entity. Since SPC_2_ reports on HDL1-3 subfractions, and is the most intense of the SPC peaks, the total integral will be driven by this relationship, which has a negative correlation with ageing, obesity and is higher in women, whereas SPC_1_ and SPC_3_ are slightly higher in men and do not show a strong relationship with BMI. Combining the information afforded by both the Glyc and SPC signals provides a 5-member inflammatory panel that can be accessed robustly and efficiently using a diffusion-edited pulse sequence with an acquisition time of around 4 minutes per sample and requires no special software for peak integration. Thus, there is potential to apply this panel to various acute and chronic states of inflammation, taking into account the need to adjust for sex, age and BMI.

## Conclusions

We measured and reported on the associations between sex, age and BMI with two acetylated glycoprotein signals (GlycA and GlycB) and the three sub-regions of the SPC signal, SPC_1_, SPC_2_ and SPC_3_. While the impact of age and BMI on the composite SPC signal has been reported in previous publications, we show strong differences in association between the 3 subcomponents of the SPC signal and age, sex, and BMI. Within a healthy population we find no significant correlation with age, however women have significantly higher SPC_2_ across all age strata. In relation to SPC and BMI, there were significant sex differences across the BMI classes of healthy weight, overweight and obese for both SPC_2_ and SPC_3_ while sex differences in SPC_1_ were only apparent in the obese BMI category. Overall SPC_2_ reduced with increasing BMI reflecting the inverse relationship between the HDL phospholipid subfraction 1–3 with obesity. SPC_1_ and SPC_3_ were less affected by changes in BMI. However, there are several limitations within this study. Firstly, the cohorts within this study are predominantly white, therefore further investigation is warranted in different ethnic groups. Secondly, the number of participants is reduced for under 40 years of age in comparison to over 40 years. However, these findings confirm the independent biological relevance of all 3 SPC subregions and suggest that the composite SPC signal should not be used as an inflammatory marker on its own. BMI was directly associated with GlycA and GlycB concentrations as previously reported. Taken together, this five-member inflammatory panel can provide greater granularity than previous NMR-derived inflammatory measures but the data from the current study suggest that models should be corrected for sex and that age and BMI should be included as covariates in models where SPC_2_ is used.

## Supporting information

S1 File (DOCX)
