# Supplementary material for: NMR spectroscopy derived plasma biomarkers of inflammation in human populations: Influences of age, sex and adiposity
Source: PLoS One. 2025 Jan 6;20(1):e0311975. doi: 10.1371/journal.pone.0311975 (PMC11703007; doi:10.1371/journal.pone.0311975)
Supplement: S1 File — (DOCX) [file pone.0311975.s001.docx]

**NMR Spectroscopy derived plasma biomarkers of inflammation in human populations: Influences of age, sex and adiposity**

Samantha Lodge^1^, Reika Masuda^1^, Philipp Nitschke^1^, John P. Beilby^2^, Jennie Hui^3^, Michael Hunter^4^, Bu B. Yeap^5,6^, Oscar Millet^7^, Julien Wist^1,8,9^, and Jeremy K. Nicholson^1,5,10*^, Elaine Holmes^1,8*^

*^1^Australian National Phenome Center and Center for Computational and Systems Medicine, Health Futures Institute, Murdoch University, Perth, Western Australia, Australia.*

*^2^School of Biomedical Sciences, University of Western Australia, Perth, Western Australia, Australia.*

*^3^PathWest Laboratory Medicine, Queen Elizabeth II Medical Centre, Perth, Western Australia, Australia.*

*^4^School of Population and Global Health, University of Western Australia, Perth, Western Australia, Australia.*

*^5^School of Medicine, University of Western Australia, Crawley, Western Australia, Australia.*

*^6^Department of Endocrinology and Diabetes, Fiona Stanley Hospital, Murdoch, Western Australia, Australia.*

*^7^Precision Medicine and Metabolism Laboratory, CIC bioGUNE, Parque Tecnológico de Bizkaia, Derio, Spain.*

*^8^Department of Metabolism, Digestion and Reproduction, Faculty of Medicine, Imperial College London, London, United Kingdom.*

*^9^Chemistry Department, Universidad del Valle, Cali, Colombia.*

*^10^Institute of Global Health Innovation, Faculty of Medicine, Imperial College London, London, United Kingdom.*

*Corresponding authors:

[Jeremy.Nicholson@murdoch.edu.au](mailto:Jeremy.Nicholson@murdoch.edu.au) and [j.nicholson@imperial.ac.uk](mailto:j.nicholson@imperial.ac.uk) (JKN)

Elaine.Holmes@Murdoch.edu.au and [elaine.holmes@imperial.ac.uk](mailto:elaine.holmes@imperial.ac.uk) (EH)

**Key words:** NMR spectroscopy, metabolic phenotyping, inflammation, Supramolecular Phospholipid Composite, SPC, glycoproteins, Healthy ranges, age, BMI.

Table of contents – Supplementary Data

| **Section 1 - Cohort Demographic Data** | **Page** |
| --- | --- |
| Table S1 - Full cohort demographic data for the Population Cohort (Busselton Study) at baseline. | S3 |
| Table S2 - Demographic data for the Basque cohort. | S3 |
| **Section 2 - Characterization of Healthy Participants** |  |
| Table S3 - P-values between the genders within each age group that are shown in figure 1. | S4 |
| Figure S1 - Box plot showing age and gender differences in healthy individuals of SPC. | S4 |
| Figure S2 - Boxplots showing the age distribution of the healthy men only. | S5 |
| Figure S3 - Boxplots showing the age distribution of the healthy women only. | S5 |
| Table S4 - Median and ranges of the inflammatory markers at each range for the healthy men only. | S6 |
| Table S5 - Median and ranges of the inflammatory markers at each range for the healthy women only. | S6 |
| **Section 3 – Perturbations caused by BMI** |  |
| Table S6 - P-values between the sexes within each BMI class that are shown in figure 2A. | S6 |
| Table S7 - Median and ranges of the inflammatory markers at each BMI range for the men and women. | S7 |
| Figure S4 - Boxplots showing the BMI distribution of the men only. | S7 |
| Figure S5 - Boxplots showing the BMI distribution of the women only. | S8 |
| Figure S6 - Boxplot showing the sex differences at each BMI class for SPC. | S8 |
| Figure S7 - Boxplots showing the BMI distribution of men and women for SPC. | S9 |
| Table S8 – P-values between the BMI classes as shown in figure 2B. | S9 |
| Figure S8 – Linear models of SPC_2_ versus different HDL phospholipid subfractions | S10 |

##

##

## **Table S1 - Full cohort demographic data for the population cohort (Busselton Study) at baseline.**

|  | **Whole cohort**  **(n=1976)** | **Women**  **(n=1071)** | **Men**  **(n=905)** |
| --- | --- | --- | --- |
| Age, years (SD) | 57.9 (±5.38) | 57.5 (±5.32) | 58.3 (±5.42) |
| BMI, kg/m^2^ (SD) | 27.3 (±4.81) | 26.7 (±5.38) | 27.7 (±4.00) |
| Diabetes | 163 (8.2%) | 91 (8.5%) | 72 (8.0%) |
| Cardiovascular disease | 125 (6.3%) | 54 (5.0%) | 71 (7.8%) |
| Hypertension | 827 (41.9%) | 416 (38.8%) | 411 (45.4%) |
| Hyperlipidemia | 703 (35.6%) | 375 (35.0%) | 328 (36.2%) |
| Cancer | 188 (9.5%) | 122 (11.4%) | 66 (7.3%) |
| Osteoporosis | 80 (4.0%) | 63 (5.9%) | 17 (1.9%) |
| Neurological conditions | 92 (4.7%) | 57 (5.3%) | 35 (3.9%) |
| Rheumatological conditions | 245 (12.4%) | 104 (9.7%) | 141 (15.6%) |
| Gastrointestinal conditions | 401 (20.3%) | 247 (23.1%) | 154 (17.0%) |
| Respiratory conditions | 437 (22.1%) | 271 (25.3%) | 166 (18.3%) |
| Mental health conditions | 264 (13.4%) | 179 (16.7%) | 85 (9.4%) |
| Endocrine conditions | 289 (14.6%) | 255 (23.8%) | 34 (3.8%) |
| Genitourinary conditions | 71 (3.6%) | 36 (3.4%) | 35 (3.9%) |
| Musculoskeletal conditions/chronic pain/chronic fatigue | 240 (12.1%) | 140 (13.1%) | 100 (11%) |
| Allergic/atopic conditions | 202 (10.2%) | 122 (11.4%) | 80 (8.8%) |

## **Table S2 - Demographic data for the Basque cohort.**

|  | **Healthy Controls (n=80)** |
| --- | --- |
| Sex, Men | 43 (53.75%) |
| Age, years (SD) | 48.00 (±12.62) |
| BMI, kg/m^2^ (SD) | 24.13 (±4.51) |

**Table S3 - P-values between the sexes within each age group (years) that are shown in Figure 1.** Significant adjusted *p*-values are highlighted in bold.

| **Spectral Variable** | **20<30 years**  ***p*-values** | **30<40 years** | **40<50 years** | **50<60 years** | **60<70 years** |
| --- | --- | --- | --- | --- | --- |
| SPC_1_ | 0.689 | **0.020** | 0.169 | **0.020** | 0.783 |
| SPC_2_ | **0.018** | **0.013** | **3.85x10^-8^** | **7.87x10^-12^** | **3.41x10^-10^** |
| SPC_3_ | 0.607 | 0.662 | 0.412 | **5.00x10^-5^** | 0.567 |
| GlycA | 0.760 | **0.043** | **0.011** | 0.070 | 0.529 |
| GlycB | 0.689 | 0.852 | 0.120 | 0.240 | 0.181 |
| SPC/Glyc | 0.066 | 0.059 | **4.08x10^-6^** | **5.64x10^-6^** | **9.38x10^-7^** |


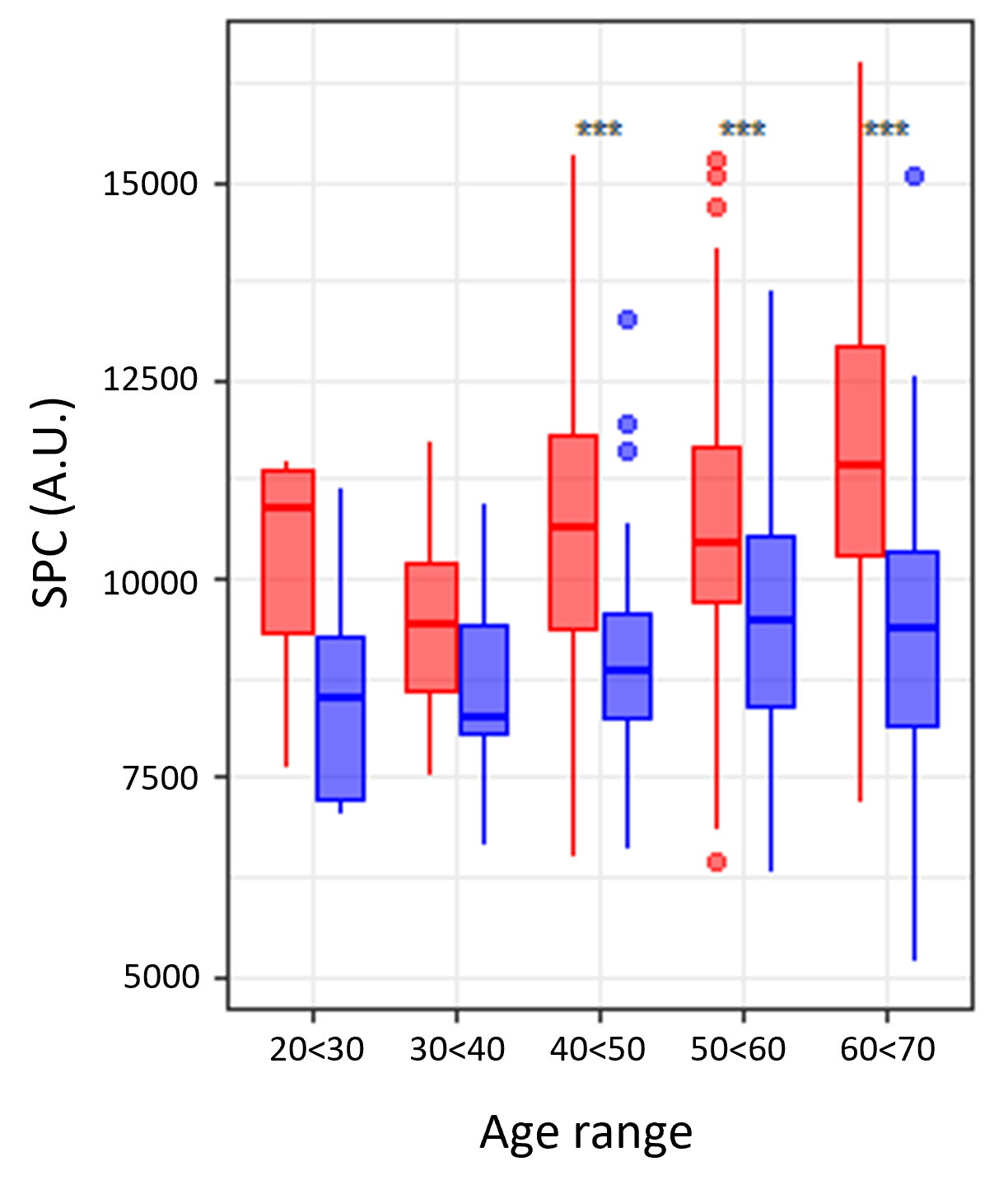


**Figure S1 - Box plot showing age and gender differences in healthy individuals of total SPC. Only the significant differences between men and women at each age range are highlighted using the *p*-value (**p*<0.05; ***p*<0.01; ****p*<0.001).**

**
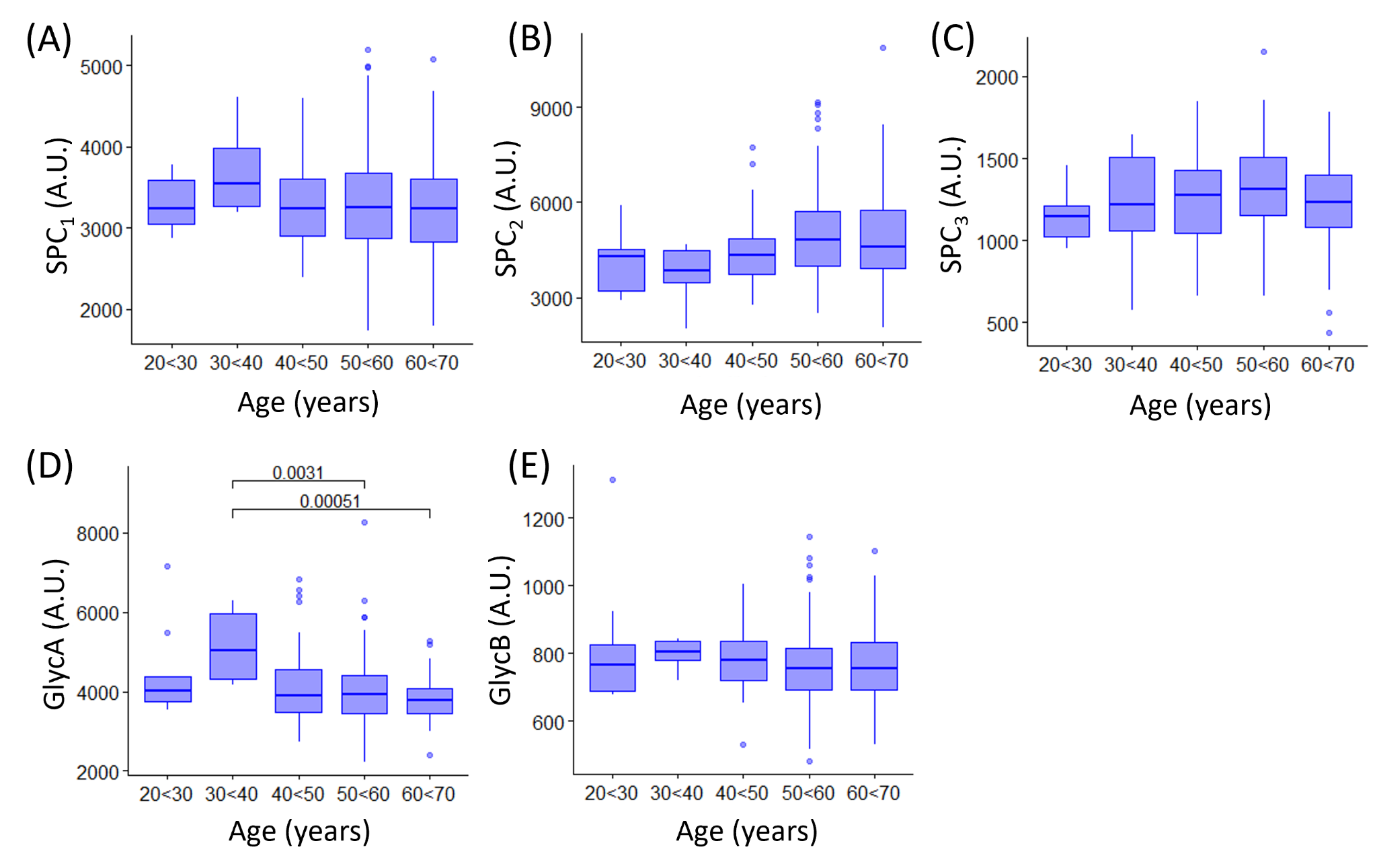
**

**Figure S2 - Box Plots showing the age distribution (years) of the healthy men only.** Only significant adjusted *p*-values are shown.


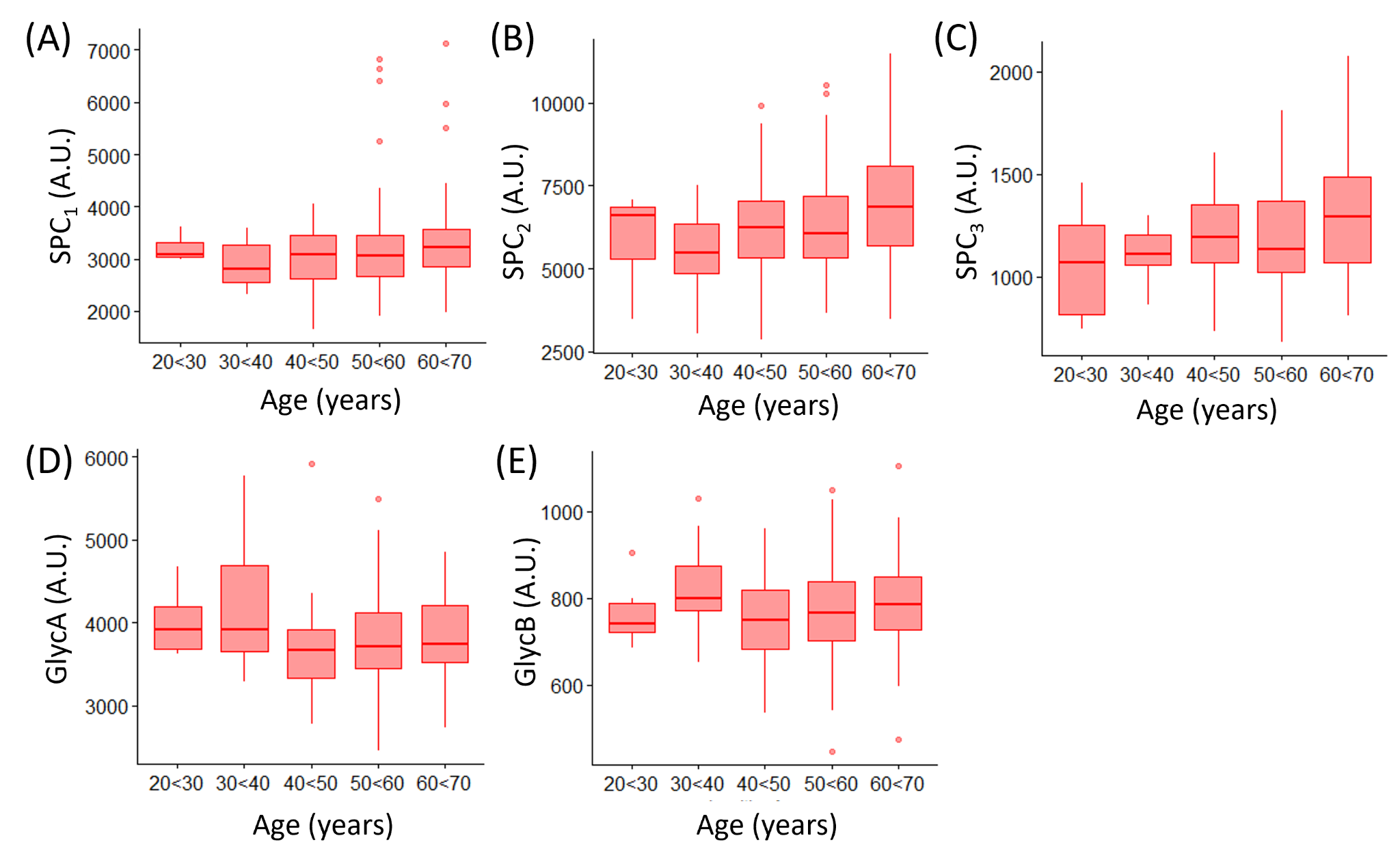


**Figure S3 - Box plots showing the age distribution of the healthy women only.** There were no significant adjusted *p*-values.

**Table S4 - Median and ranges of the inflammatory markers at each age range (years) for healthy men only.**

| **Spectral Variable**  **(A.U.)** | **20<30 years**  (n=9) | **30<40 years**  (n=6) | **40<50 years**  (n=42) | **50<60 years**  (n=133) | **60<70 years**  (n=81) |
| --- | --- | --- | --- | --- | --- |
| GlycA | 3892.46 [3541.02-4386.19] | 5143.50  [4156.85-6300.12] | 4119.09  [2735.28-6555.78] | 3947.22  [2234.40-6279.03] | 3792.90  [2414.29-5263.47] |
| GlycB | 740.51  [676.66-823.91] | 798.68  [719.30-842.79] | 781.08  [530.74-1004.29] | 754.76  [482.83-1083.19] | 766.18  [531.18-1101.63] |
| SPC_1_ | 3296.99  [2868.07-3770.02] | 3697.85  [3192.77-4608.31] | 3233.38  [2395.00-4590.95] | 3293.35  [1741.95-5186.87] | 3213.42  [1800.00-5072.64] |
| SPC_2_ | 4335.30  [3194.86-5886.52] | 3736.61  [2031.04-4674.72] | 4466.63  [2752.26-7736.55] | 4976.54  [2512.64-9137.85] | 4876.08  [2033.76-10864.24] |
| SPC_3_ | 1125.71  [948.06-1460.32] | 1210.73  [573.99-1643.10] | 1269.91  [799.16-1845.44] | 1326.53  [664.35-2149.95] | 124..83  [441.05-1781.16] |

**Table S5 - Median and ranges of the inflammatory markers at each age range for the healthy women only.**

| **Spectral Variable** | **20<30 years**  **(n=6)** | **30<40 years**  **(n=8)** | **40<50 years**  **(n=32)** | **50<60 years**  **(n=106)** | **60<70 years**  **(n=55)** |
| --- | --- | --- | --- | --- | --- |
| GlycA | 4004.12  [3631.95-4673.69] | 4193.81  [3293.23-5768.42] | 3663.69  [2772.95-5914.60] | 3770.59  [2463.86-5500.99] | 3845.29  [2730.11-4857.27] |
| GlycB | 764.60  [685.37-903.74] | 828.07  [651.34-1031.08] | 743.25  [536.19-961.74] | 771.52  [447.17-1049.43] | 789.07  [474.83-1104.67] |
| SPC1 | 3197.75  [3000.77-3606.38] | 2920.45  [2328.73-3591.18] | 3039.11  [1664.84-4048.87] | 3155.86  [1914.27-6815.98] | 3348.56  [1975.29-7119.76] |
| SPC2 | 5937.32  [3469.81-7094.91] | 5435.09  [3048.18-7527.02] | 6403.58  [3888.15-9926.78] | 6303.25  [3666.50-10547.08] | 6926.96  [3491.72-11478.21] |
| SPC3 | 1062.52  [746.81-1456.40] | 1107.18  [859.91-1295.02] | 1209.25  [732.78-1603.50] | 1175.95  [681.98-1812.27] | 1299.06  [808.85-2074.01] |

**Table S6 - *P*-values between the sexes within each BMI class that are shown in figure 2A.** Significant adjusted *p*-values are highlighted in bold.

| **Spectral Variable** | **Healthy weight**  **BMI 18.5<25 kg/m^2^** | **Overweight**  **BMI 25<30 kg/m^2^** | **Obese**  **BMI >30 kg/m^2^** |
| --- | --- | --- | --- |
| SPC_1_ | 0.43 | 0.64 | **3.67x10^-4^** |
| SPC_2_ | **3.46x10^-43^** | **3.33x10^-79^** | **2.15x10^-64^** |
| SPC_3_ | **9.39x10^-3^** | **5.72x10^-3^** | **1.09x10^-5^** |
| GlycA | 0.06 | 0.43 | 0.11 |
| GlycB | **0.01** | **6.96x10^-6^** | **6.84x10^-12^** |
| SPC/Glyc | **5.53x10^-21^** | **2.82x10^-40^** | **1.18x10^-27^** |

**Table S7 - Median and ranges of the inflammatory markers at each BMI range (kg/m^2^) for men and women.**

| **Spectral Variable** | **Male**  **18.5<25.0** | **Female**  **18.5<25.0** | **Male**  **25.0<30.0** | **Female**  **25.0<30.0** | **Male**  **>30** | **Female**  **>30** |
| --- | --- | --- | --- | --- | --- | --- |
| GlycA | 3785.54  [2414.29-6704.02] | 3851.91  [1761.91-10137.36] | 4311.41  [2234.40-145.60.49] | 4222.44  [2319.33-9436.51] | 4521.45  [2787.35-8587.46] | 4543.45  [2772.39-7911.09] |
| GlycB | 758.49  [482.83-1376.71] | 777.37  [447.17-1346.43] | 807.72  [379.50-1309.55] | 831.18  [450.88-1219.72] | 852.38  [438.33-1384.20] | 898.08  [547.45-1266.63] |
| SPC_1_ | 3156.63  [1755.53-6710.71] | 3183.90  [1191.52-7862.14] | 3279.83  [1522.25-6222.09] | 3298.97  [1732.90-6413.79] | 3221.94  [1745.08-5870.73] | 3362.88  [1773.11-8055.28] |
| SPC_2_ | 5123.65  [2124.82-10864.24] | 6837.86  [1888.15-14421.17] | 4609.95  [1839.04-11916.27] | 6136.34  [2341.34-11986.60] | 4085.73  [1802.10-8438.08] | 5502.43  [2165.62-12876.15] |
| SPC_3_ | 1266.68  [529.28-2760.97] | 1319.02  [372.91-2370.54] | 1306.41  [414.58-2479.44] | 1356.99  [482.33-3474.894] | 1226.61  [422.82-2627.86] | 1324.70  [540.05-2610.58] |


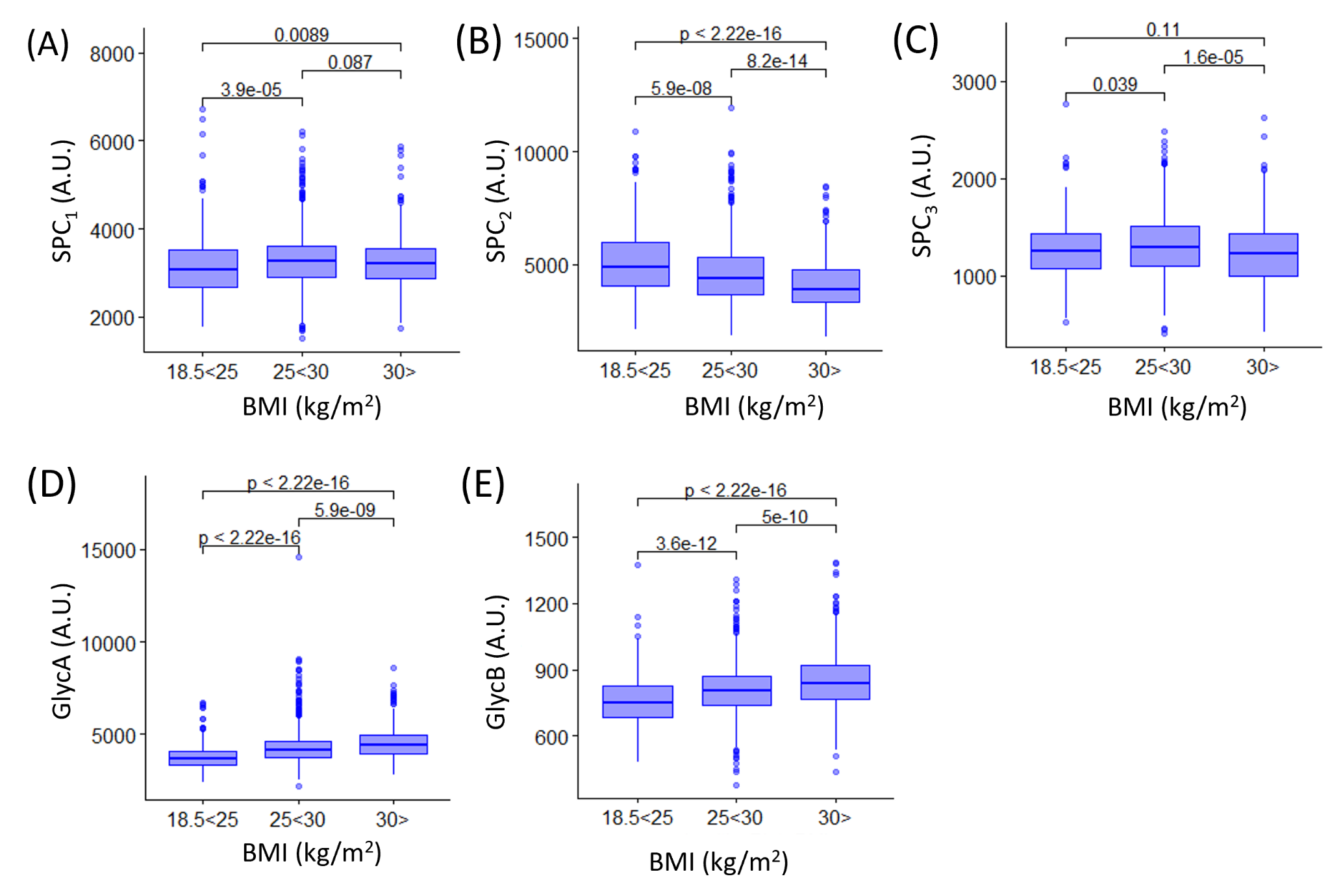


**Figure S4 - Boxplots showing the BMI distribution (kg/m^2^) of the men only for (A) SPC_1_ (B) SPC_2_ (C) SPC_3_ (D) GlycA, (E) GlycB**. *P*-values are shown.


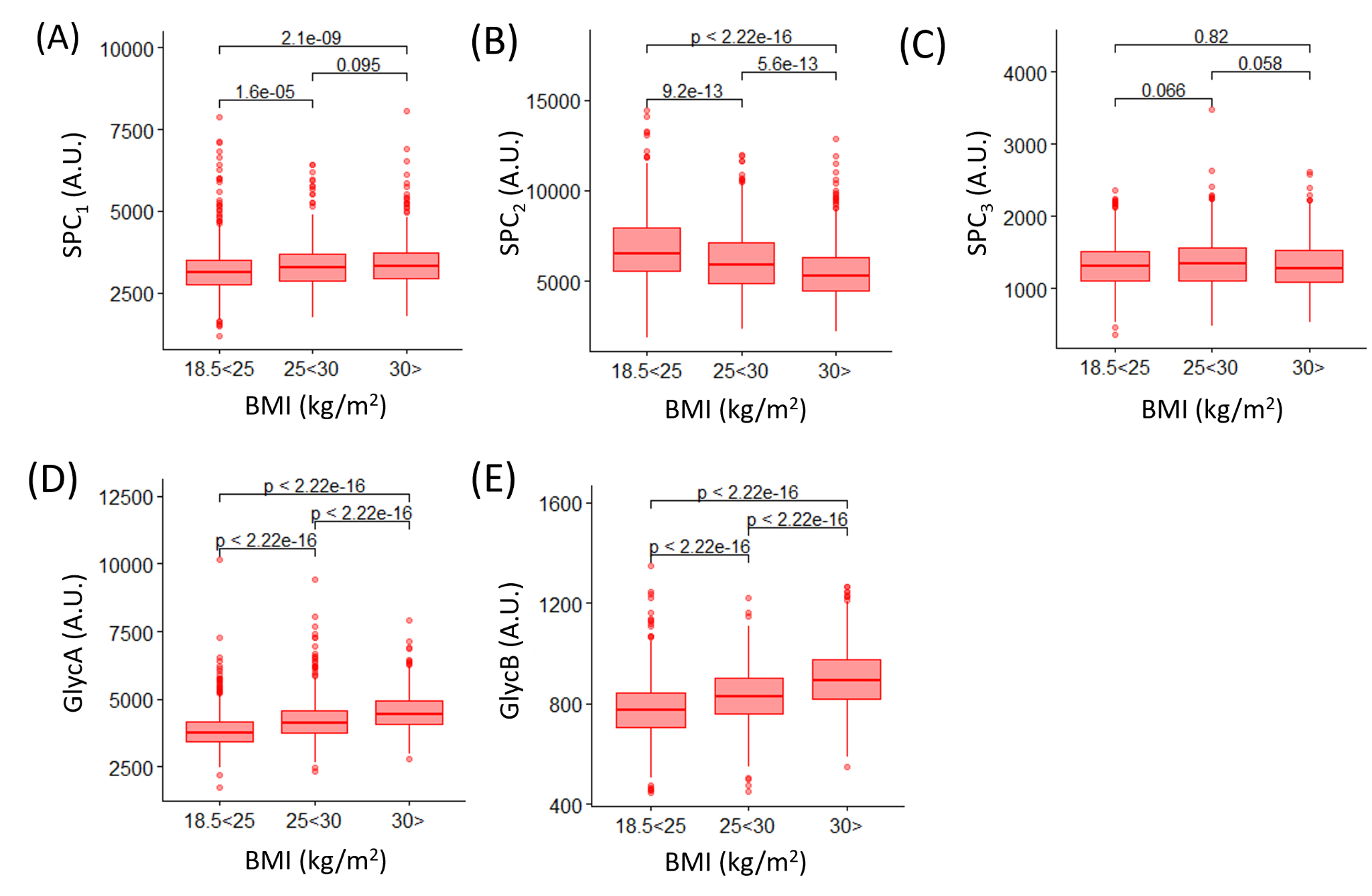


**Figure S5 - Boxplots showing the BMI distribution (kg/m^2^) of the women only for (A) SPC_1_ (B) SPC_2_ (C) SPC_3_ (D) GlycA, (E) GlycB**. *P*-values are shown.


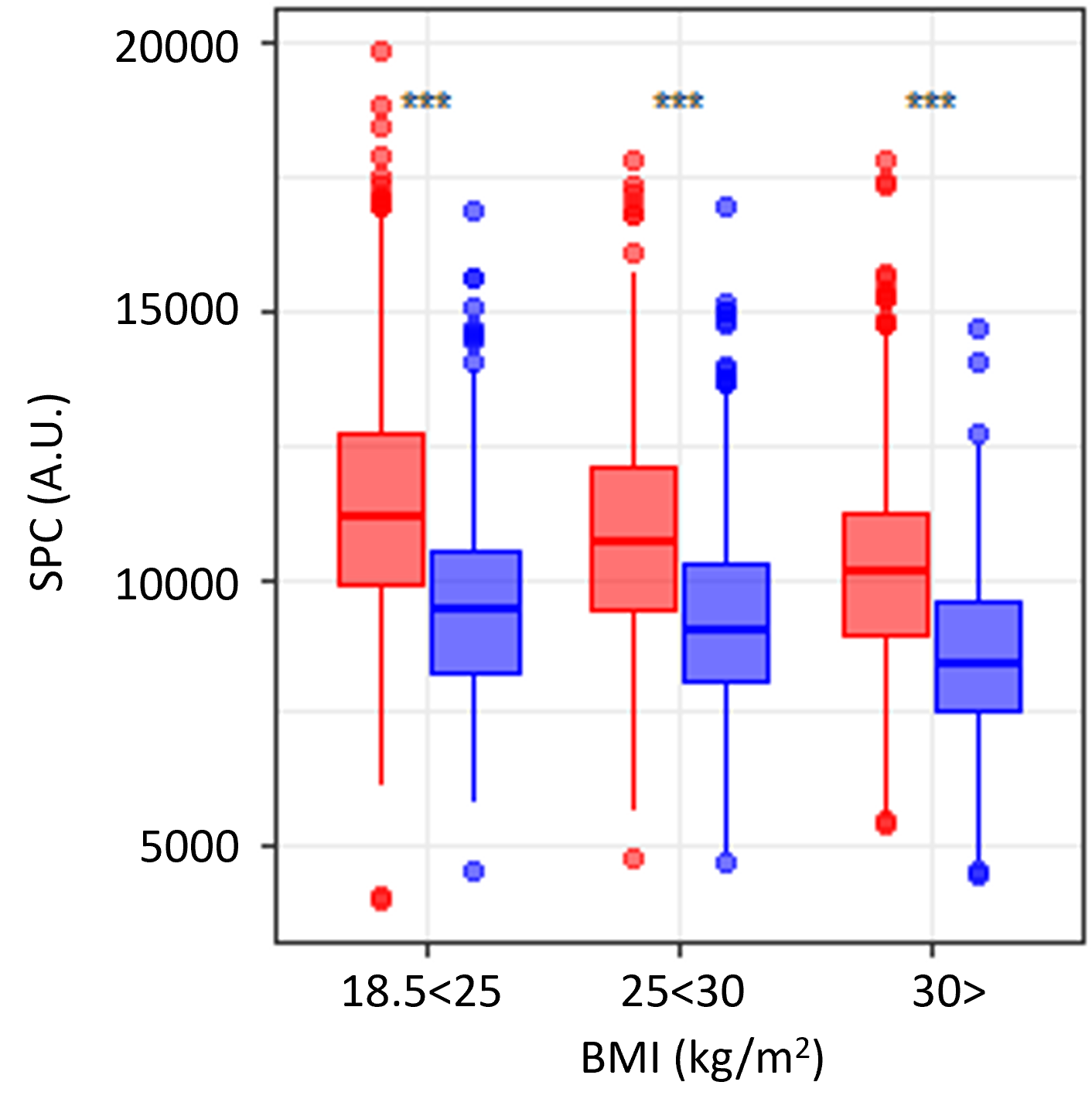


**Figure S6 - Box plot showing the sex differences for each BMI class for SPC (n=3936).** 18.5<25 *p*-value=1.19x10^-35^, 25<30 *p*-value=1.26x10^-60^, >30 *p*-value=2.49x10^-53^.


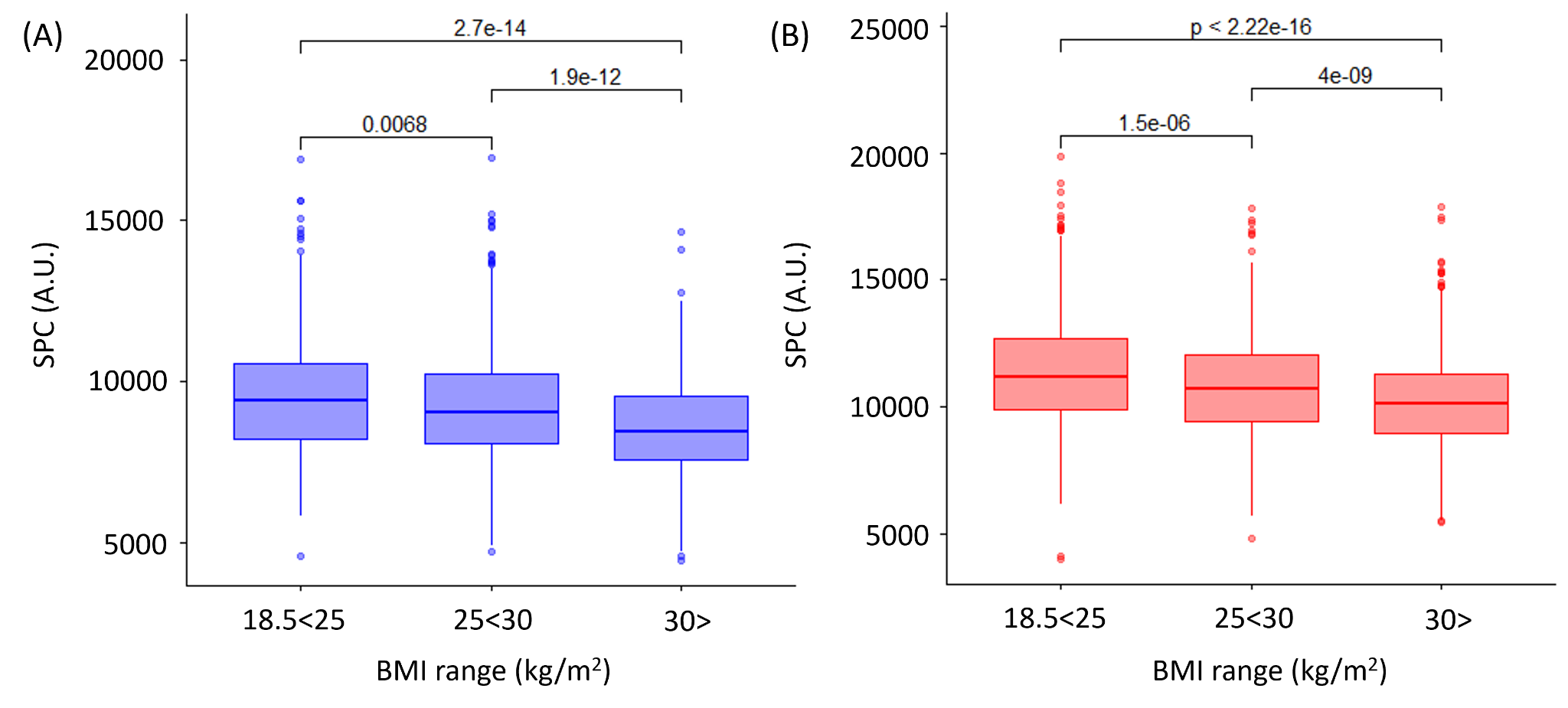


**Figure S7 - Boxplots showing the BMI distribution of the (A) men and (B) women for SPC.** *P*-values are shown in plot.

**Table S8 - *P*-values between the BMI classes as shown in figure 2B.** Significant adjusted *p*-values are highlighted in bold.

| **Spectral Variable** | **Healthy weight vs overweight**  **(BMI 18.5<25 vs 25<30 kg/m^2^)** | **Healthy weight vs obese**  **(BMI 18.5<25 vs >30 kg/m^2^)** | **Overweight vs obese**  **(BMI 25<30 vs >30 kg/m^2^)** |
| --- | --- | --- | --- |
| SPC_1_ | **6.70x10^-12^** | **1.00x10^-10^** | 0.86 |
| SPC_2_ | **7.10x10^-23^** | **2.80x10^-68^** | **3.50x10^-25^** |
| SPC_3_ | **4.80x10^-3^** | 0.20 | **3.30x10^-5^** |
| GlycA | **5.10x10^-49^** | **2.20x10^-108^** | **6.60x10^-30^** |
| GlycB | **3.60x10^-30^** | **6.30x10^-86^** | **3.40x10^-34^** |
| SPC/Glyc | **1.20x10^-58^** | **4.90x10^-138^** | **8.00x10^-47^** |


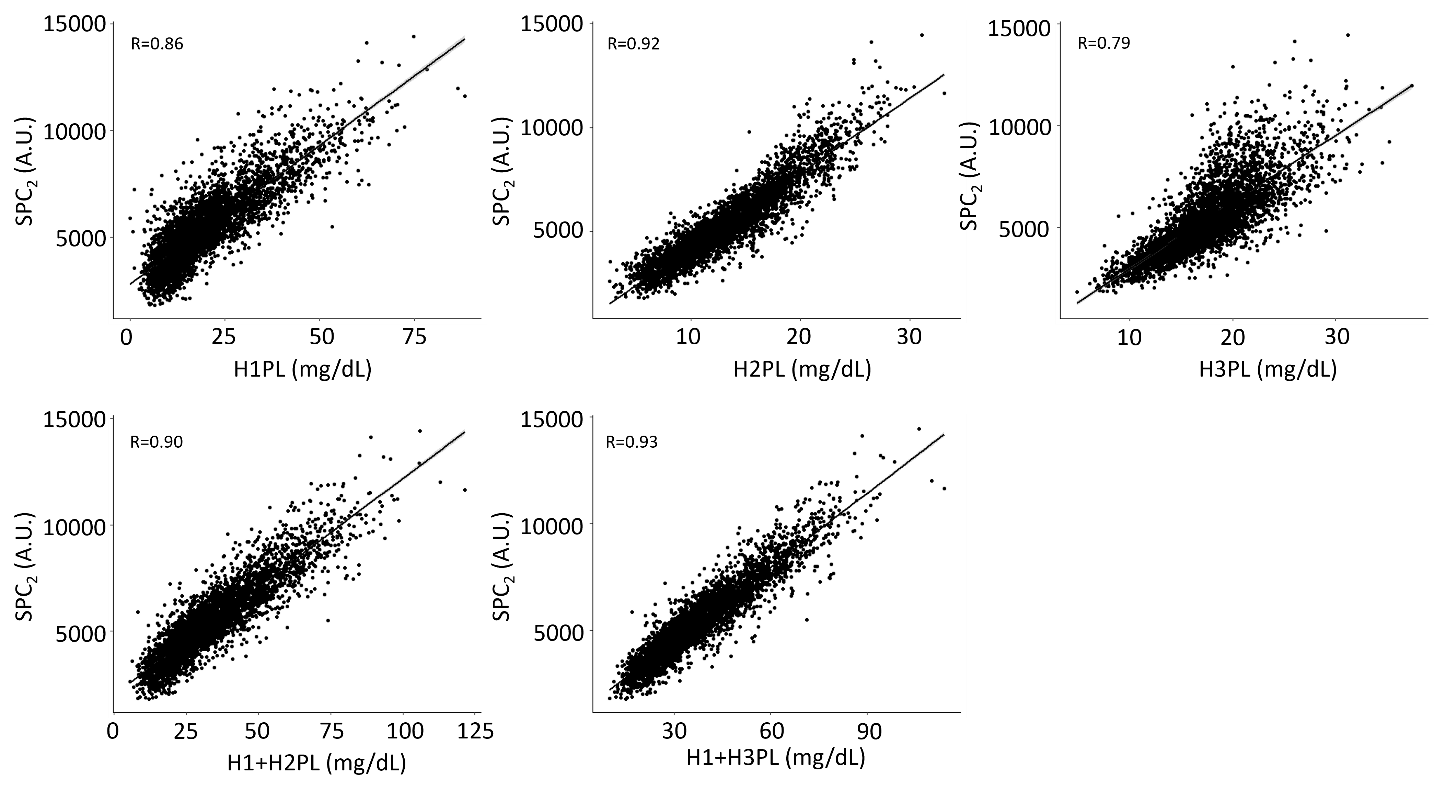


**Figure S8 – Linear models of SPC_2_ versus different HDL phospholipid subfractions. (n=4025, BHAS and Basque population included).**
